# 1670. It Is Likely Not HSV: Decreasing Empiric Acyclovir Use In Low-Risk Infants

**DOI:** 10.1093/ofid/ofad500.1503

**Published:** 2023-11-27

**Authors:** Guillermo Yepes Junquera, Jeanette Taveras, Joshua R Watson, Malak Abdel-Hadi, Guliz Erdem

**Affiliations:** Nationwide Children's Hospital, Columbus, Ohio; Nationwide Children's Hospital, Columbus, Ohio; Nationwide Children's Hospital, Columbus, Ohio; Nationwide Children's Hospital - Center for Clinical Excellence, Columbus, Ohio; Nationwide Children's Hospital, Columbus, Ohio

## Abstract

**Background:**

Neonatal HSV infection is a rare but potentially devastating disease. Institutional practices regarding which neonates must be treated empirically with acyclovir varies. While some centers elect to treat all neonates presenting with fever or hypothermia, some treat only those considered at risk. Our goal was to decrease empiric acyclovir use in low-risk neonates admitted to the Infectious Disease (ID) ward.

**Methods:**

Quality improvement initiative aimed to decrease acyclovir exposure in neonates 15 to 28 days old hospitalized on the ID ward for fever/hypothermia without a focus, risk factors, or clinical signs for HSV **(Figure 1)**. Interventions included development of a risk stratification algorithm **(Figure 2)**, consensus building among Emergency Medicine and ID providers, and order set implementation. We reviewed all neonates with HSV infections for delayed acyclovir initiation as a balancing measure. The pre-study baseline period was 2019 to 2020, and the study period was 2020, to March 31, 2023. Control charts were used to assess the interventions.

**Figure 1:**
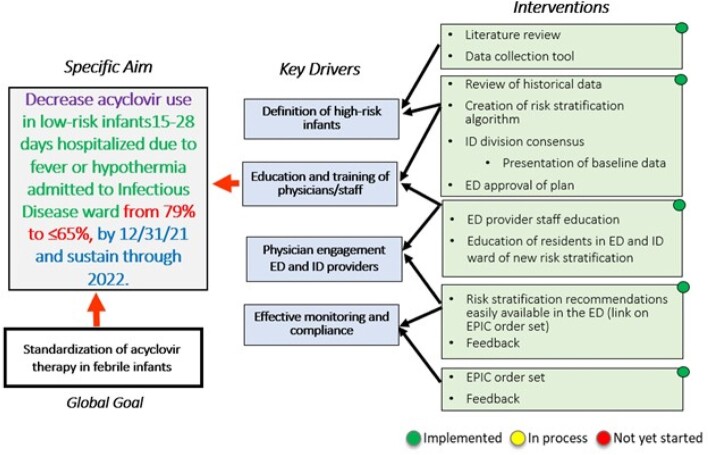
Key Driver Diagram.

**Figure 2:**
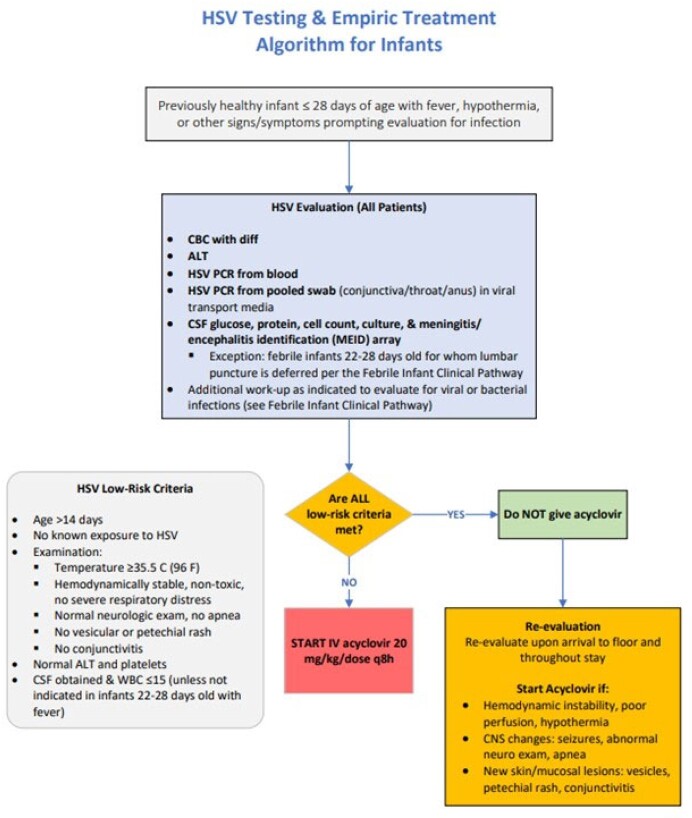
Risk stratification algorithm for HSV empiric treatment in low-risk infants.

**Results:**

The mean acyclovir use per quarter in neonates who met low risk criteria decreased from 79% during the baseline period (7/2019-6/2020) to 30% in the post-intervention period (7/2020-3/2023) (p = < 0.0001) **(Figure 3)**. Most common reasons why patients failed low risk criteria were: CSF pleocytosis in the setting of a traumatic LP, minimally abnormal lab values (slightly increased ALT, slightly decreased platelets), no LP in well-appearing patient and alternative fever etiology (URI symptoms with positive respiratory infection array). There were no cases of delayed acyclovir initiation in patients with HSV disease. Only one patient with HSV disease fell within the age range but was admitted to the ICU following cardiac arrest and appropriately started on acyclovir therapy.

**Figure 3:**
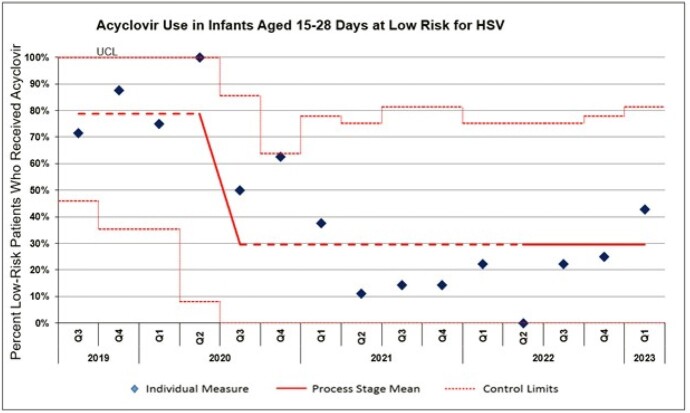
Control chart of empiric acyclovir use in low-risk infants.

**Conclusion:**

Our algorithm, targeted to limit empiric acyclovir therapy in neonates with fever/hypothermia without a focus at low risk for HSV, led to a significant decrease in acyclovir exposure sustained for > 2 years after implementation. Interestingly, it also led to decreased in acyclovir use in patients who failed low risk criteria, but this did not result in missed diagnosis of HSV disease, likely because our algorithm was conservative and HSV infection is rare.

**Disclosures:**

**All Authors**: No reported disclosures

